# Evolutionary and ecological role of extracellular contractile injection systems: from threat to weapon

**DOI:** 10.3389/fmicb.2023.1264877

**Published:** 2023-10-11

**Authors:** Clara Margot Heiman, Jordan Vacheron, Christoph Keel

**Affiliations:** Department of Fundamental Microbiology, University of Lausanne, Lausanne, Switzerland

**Keywords:** extracellular contractile injection system, tailocin, phage, phage tail like structure, explosive cell lysis, bacterial ecology, bacteria host interaction, biotechnology

## Abstract

Contractile injection systems (CISs) are phage tail-related structures that are encoded in many bacterial genomes. These devices encompass the cell-based type VI secretion systems (T6SSs) as well as extracellular CISs (eCISs). The eCISs comprise the R-tailocins produced by various bacterial species as well as related phage tail-like structures such as the antifeeding prophages (Afps) of *Serratia entomophila*, the *Photorhabdus* virulence cassettes (PVCs), and the metamorphosis-associated contractile structures (MACs) of *Pseudoalteromonas luteoviolacea*. These contractile structures are released into the extracellular environment upon suicidal lysis of the producer cell and play important roles in bacterial ecology and evolution. In this review, we specifically portray the eCISs with a focus on the R-tailocins, sketch the history of their discovery and provide insights into their evolution within the bacterial host, their structures and how they are assembled and released. We then highlight ecological and evolutionary roles of eCISs and conceptualize how they can influence and shape bacterial communities. Finally, we point to their potential for biotechnological applications in medicine and agriculture.

## Introduction

Bacteriophages (or phages), the viruses that infect bacterial cells, are highly efficient predators. They take advantage of their host by adopting two main infection strategies, namely efficient productive replication or persistent dormancy (non-productive), depending on the environment and host density (Chevallereau et al., 2021). In productive replication, phages use their host bacteria as viral factories to replicate themselves. Once the viral progeny is fully assembled, it is either released through the abrupt lysis of the bacterial host (lytic cycle) or continuously without lysing the host cell (chronic cycle; Salmond and Fineran, 2015; Chevallereau et al., 2021; Correa et al., 2021). Conversely, during dormancy, the viral genetic content is integrated into the genome of the bacterial host, becoming a prophage (Lwoff et al., 1950; Feiner et al., 2015; Salmond and Fineran, 2015; Chevallereau et al., 2021; lysogenic cycle). A prophage can remain dormant until it is spontaneously activated or until its host encounters a stress that damages DNA and triggers the bacterial SOS response, stimulating the production of the viral progeny and the lysis of the host cell (Howard-Varona et al., 2017). Although phages had been classified into distinct categories, their infection strategies now are rather seen as a continuum depending on the environment and host density (Chevallereau et al., 2021).

**Figure 1:**
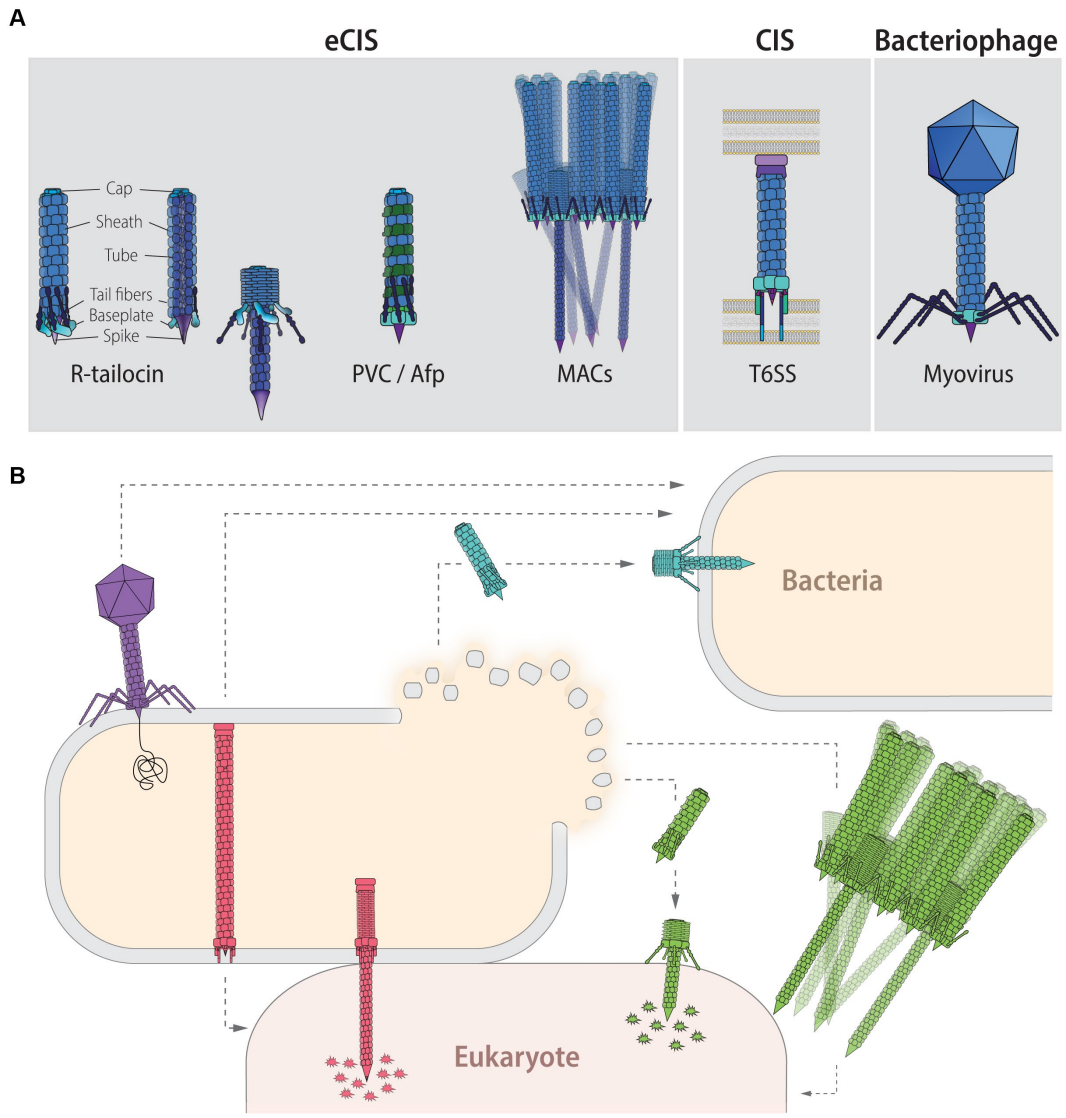
Contractile injection systems (CISs): classification, targets and function. **(A)** CISs are thought to be evolved from the contractile tail of *Myoviridae* phages and can be classified into two main groups: extracellular CISs (eCISs) and cell wall-anchored type VI secretion systems (T6SSs). Extracellular CISs encompass R-tailocins (left, extended state; right contracted state; colors indicate the different building blocks) as well as various phage tail-like structures such as the *Photorhabdus* virulence cassettes (PVCs), the antifeeding prophages (Afps), and the metamorphosis-inducing contractile structures (MACs). Sizes of eCISs range between 80 and 180 nm, for R-tailocins, Afps and PVCs, while MACs form larger arrays which have been reported to range up to 920 nm, formed out of individual components of about 310 nm. **(B)** Phages (purple) directly interact with a host bacterial cell by injecting their genetic content. T6SSs (red) are assembled in the producing cell and upon contact with a target cell, either bacterial or eukaryotic, the device contracts and injects toxic effectors into the prokaryotic or eukaryotic prey. Extracellular CISs are released upon lysis of the producing cell. R-tailocins (light blue) have only been found to target other bacteria by puncturing and destabilizing their membrane without injecting effectors, while protein-translocating eCISs like PVCs, Afps and MACs (green) target eukaryotic cells by injecting eCIS-associated toxins (EATs). For MACs, only part of the star-shaped structure formed by the assembly of a large number of eCISs is shown.

**Figure 2:**
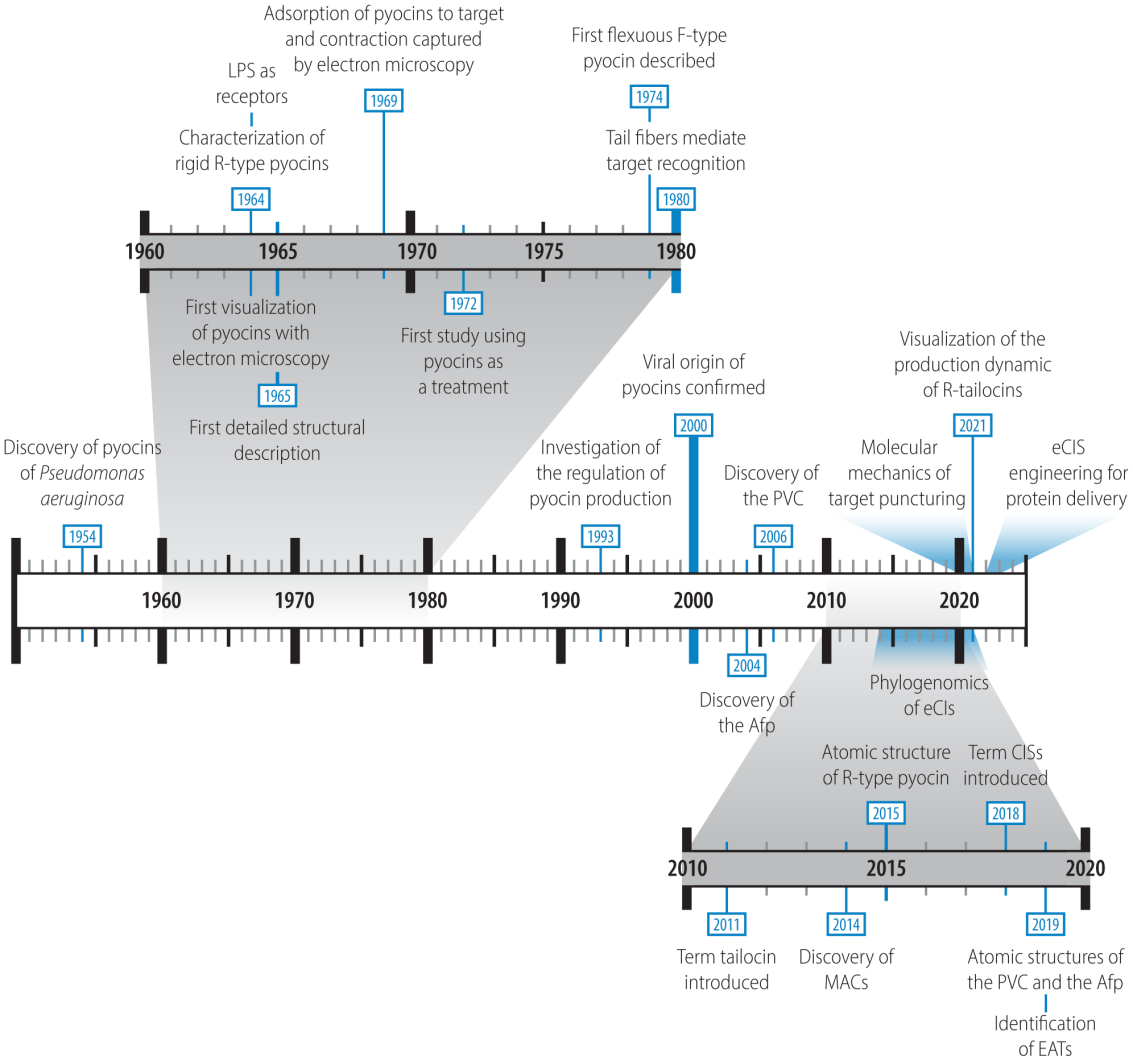
Timeline highlighting the main extracellular contractile systems (eCISs) discoveries. The relevant references for these discoveries are listed in Supplementary Table 1.

It may seem that bacteria are the victims, with no benefit from interacting with these viral predators. However, this assumption would not take into account the fact that prophages can be harnessed by their host. In fact, some of these prophages remain dormant and through the evolution of the genome of their host, they are subject to mutations, genomic rearrangements and degradation of their genome, which makes them no longer inducible or able to self-replicate (Canchaya et al., 2003). Although they are no longer independent entities, these evolved dormant viruses can be beneficial to their host. For example by becoming cryptic (Canchaya et al., 2003), they may benefit the bacterium by contributing genes that increase bacterial fitness (Wang et al., 2010) or provide protection from other viral predators (Bobay et al., 2014; Da Silva Duarte et al., 2019; Ragunathan and Vanderpool, 2019; Hampton et al., 2020; Correa et al., 2021; Patel and Maxwell, 2023). Indeed, genetic elements derived from cryptic prophages have been shown to contribute to heightened antibiotic resistance (Wang et al., 2010), while others lead to superinfection exclusion against similar phage predators (Ofir and Sorek, 2018; Da Silva Duarte et al., 2019; Ragunathan and Vanderpool, 2019; Hampton et al., 2020; Correa et al., 2021; Patel and Maxwell, 2023). Furthermore, some of these prophages lose the genes that specify replication and capsid formation, generating particles that resemble “headless” phage particles also referred to as phage tail-like particles (Bobay et al., 2014). Prophage ancestors belonging to the *Myoviridae* family of the *Caudovirales* order can give rise to structures with a contractile tail, now commonly called contractile injection systems (CISs; Taylor et al., 2018; Figures 1, 2; Supplementary Table 1), which can be repurposed as weapons capable of killing competing bacteria or affecting eukaryotic cells.

The CISs can be classified into two distinct groups: the intracellular, cell wall-anchored type VI secretion systems (T6SSs) and the extracellular contractile injection systems (eCISs; Sarris et al., 2014; Taylor et al., 2018; Patz et al., 2019). For the sake of simplicity, in this review a nomenclature in which eCISs encompass the R-type tailocins (hereafter referred to as R-tailocins) and various phage tail-like protein translocation structures (Figure 1) is used. The best-known CISs are the T6SSs, which act primarily against bacteria but are also involved in interactions with eukaryotic cells. T6SSs are intricate syringe-like complexes docked into the cytoplasmic membrane of the attacking bacteria that inject toxic and lytic effectors into target cells where they disrupt basic cellular components such as cell wall and cytoskeletal structures and nucleic acids (Ho et al., 2014; Russell et al., 2014; Durand et al., 2015; Cianfanelli et al., 2016; Cherrak et al., 2019; Coulthurst, 2019; Wang et al., 2019; Hernandez et al., 2020; Figure 1). Conversely to the T6SSs, eCISs are extracellular phage tail-like weapons that are assembled in the cytoplasm and are only released and active upon lysis of the producing bacterial cell (Patz et al., 2019; Figure 1). Extracellular CISs comprise a wide variety of structures, including the contractile R-tailocins, the antifeeding prophages (Afps) of *Serratia entomophila* (Hurst et al., 2004), the *Photorhabdus* virulence cassettes (PVCs; Yang et al., 2006), and the metamorphosis-associated contractile structures (MACs) of *Pseudoalteromonas luteoviolacea* (Shikuma et al., 2014; Figure 1). Afps, PVCs, and MACs have the capacity to transport effector proteins with toxic activities (Hurst et al., 2004; Heymann et al., 2013; Sarris et al., 2014; Ericson et al., 2019; Vlisidou et al., 2019; Geller et al., 2021; Wang et al., 2022) whereas R-tailocins are not known to carry harmful cargos (Patz et al., 2019; Figure 1). Some eCIS loci, specifically those related to eCIS that transport toxic effectors, have been further classified based on their genetic content and organization separating them into two main lineages with six distinct clades (Ia, Ib, and IIa–IId; Chen et al., 2019). Afps and PVCs belong to subtype Ia, while MACs belong to the subtype Ib (Chen et al., 2019). These different clades are characterized by syntenic differences and subtype-specific genes (Chen et al., 2019).

As the T6SSs have already been extensively reviewed (Ho et al., 2014; Russell et al., 2014; Durand et al., 2015; Cianfanelli et al., 2016; Cherrak et al., 2019; Coulthurst, 2019; Wang et al., 2019; Hernandez et al., 2020), this review will focus specifically on the eCISs, with an emphasis on the R-tailocins. First, we give a synopsis of the history of their discovery. We then look at their viral origins and summarize what is currently known about their genetics, their structures and their mechanisms of action. Furthermore, we provide insight into the ecological and evolutionary roles of eCISs focusing on how they shape bacterial communities, how they mediate interactions with a eukaryotic host, and how they influence bacterial evolution. Finally, we illustrate how these particles can be harnessed for biotechnological applications.

## A brief history of eCISs

Although the interest for eCISs is rapidly expanding, with recent bioinformatic studies emphasizing their wide distribution and diversity among Gram-negative and Gram-positive bacteria as well as archaea (Sarris et al., 2014; Chen et al., 2019; Geller et al., 2021), the discovery of these structures along with knowledge about their ultrastructure and the mechanics of their functioning is not new. A brief history of discoveries related to eCISs is provided below. The timeline of discoveries is summarized in Figure 2 and relevant references are listed in Supplementary Table 1. François Jacob was the first who reported about tailocins in 1954 (Jacob, 1954). In his study, he described an “antibiotic substance” retrieved from a strain of *Pseudomonas aeruginosa* (identified as *Pseudomonas pyocyanea* at the time; Jacob, 1954). He found that this substance could be induced by mutagenic agents such as ultraviolet light (UVs) and that it operated upon adsorption to the membrane of a sensitive cell (Jacob, 1954). Although this product differed from conventional antibiotics in its activity, it resembled the previously discovered toxic colicins of *Escherichia coli* strains (Gratia, 1932; Fredericq, 1953; Jacob, 1954). By analogy to these, Jacob named them “pyocins,” a term still used today to describe the tailocins harbored by *P. aeruginosa* strains. Interestingly, already then, both colicins and pyocins were compared to phages in their activity mechanism, specifically looking at the potential membrane receptors targeted on sensitive bacteria (Fredericq, 1953; Jacob, 1954). It was suggested that phages, colicins and pyocins may use similar or even the same receptors to target sensitive cells (Fredericq, 1953; Jacob, 1954). In 1958, while studying lysogeny in strains of *E. coli* K12, Arber and Kellenberger, using electron microscopy, identified UV-induced structures that appeared to be “phage-related,” including phage tail-like particles that could adsorb to the surface of cells (Arber and Kellenberger, 1958). However, it is unclear whether these structures could have been potential pyocins or simply phage structural components. A few years later, the French “Académie des Sciences” dealt with the absorption mechanisms of both colicins and pyocins at their meeting on the 24th October 1960 (Hamon and Péron, 1960). This was the beginning of the appeal of these structures.

In the early 1960s, the interest for these intriguing particles shifted from Europe to Asia, where research groups in Japan began to study them (Homma and Suzuki, 1961a,b, 1964; Homma et al., 1962; Kageyama and Egami, 1962; Ikeda et al., 1964; Kageyama, 1964; Kageyama et al., 1964). Led mainly by Kageyama and colleagues they described the first R-type pyocin (R-pyocin) as a proteinaceous rod-like rigid structure resembling a phage tail, which they purified from *P. aeruginosa* strain R in their three-part “Studies of a pyocin” (Ikeda et al., 1964; Kageyama, 1964; Kageyama et al., 1964). They were able to induce the structures with DNA damaging agents such as UVs and the antibiotic mitomycin C and visualize purified R-pyocin particles by electron microscopy (Kageyama, 1964). Following in the steps of their European colleagues, Kageyama and colleagues suggested that R-pyocins, because of their similarity to phage tails, might be “defective lysogenic phages” that are unable to synthesize DNA (Ikeda et al., 1964; Kageyama, 1964; Kageyama et al., 1964). Moreover, they discovered a R-pyocin-associated lytic enzyme that is induced at the same time as the R-pyocin. They suggested that, analogous to phages, this enzyme plays a role in the lysis of the R-pyocin-producing bacteria (Kageyama et al., 1964). In addition, the first detailed structure characterization was performed in 1965, again emphasizing the resemblance of R-pyocins to phage tails (Ishii et al., 1965).

The mechanism of action was also studied during this period. Indeed, Homma and Suzuki found that lipopolysaccharides (LPS) and R-pyocins interact (Homma and Suzuki, 1964). This interaction was later confirmed when LPS were demonstrated to be a receptor of R-pyocin and various other R-tailocins (Ikeda and Egami, 1969, 1973; Köhler et al., 2010; Carim et al., 2021; Heiman et al., 2022). The attachment of R-pyocin particles to the target cell envelope was then also captured by electron microscopy (Takeya et al., 1969) and it was discovered that it was upon adsorption to the surface of a sensitive cell that the structures contracted and that this action was necessary for their toxic effect (Higerd et al., 1969; Shinomiya et al., 1975). Moreover, several studies suggested that R-pyocins kill targeted cells by disrupting their membrane (Kageyama et al., 1964; Kaziro and Tanaka, 1965; Uratani and Hoshino, 1984).

Interest in these particles continued to grow in the 1970s and 1980s as more R-pyocins became identified and purified (Chen and Tai, 1972; Garcia Rodriguez and Saenz Gonzalez, 1972; Shinomiya, 1972; Govan, 1974a; Al-Shibib et al., 1985; Al-Rubiee et al., 1988). Some of these R-pyocins were compared with each other and the tail fibers were identified as the component involved in their specificity (Ohsumi et al., 1980; Kumazaki and Ishii, 1982). The origin of R-pyocins was also explored and the relationship between R-pyocins and phages was confirmed (Shinomiya and Shiga, 1979; Shinomiya, 1984; Shinomiya and Ina, 1989; Nakayama et al., 2000). Importantly, during this period, it was found that R-type pyocins have a more efficient killing activity than headless mutants of a related phage, supporting the hypothesis that these particles are not simply defective prophages but evolved weapons (Shinomiya and Shiga, 1979). Additionally, their advantage for medical use was explored, employing pyocins for *P. aeruginosa* typing (Farmer and Herman, 1969; Rose et al., 1971; Edmonds et al., 1972; Lovrekovich et al., 1972; Jones et al., 1974; Duncan and Booth, 1975; Bruun et al., 1976; Fyfe et al., 1984; Schable et al., 1986) as well as a potential treatment against *P. aeruginosa* infections (Merrikin and Terry, 1972; Haas et al., 1974). In addition to all these findings, a new type of phage tail-like pyocin, the F-type, was discovered (Takeya et al., 1969; Govan, 1974b; Kuroda et al., 1979; Kuroda and Kageyama, 1979, 1981; Michel-Briand and Baysse, 2002; Saha et al., 2023). Unlike the rigid R-type pyocin composed of a contractile sheath and an inner core, the F-type was described as having a flexible, non-contractile tube structure without an inner core (Kuroda and Kageyama, 1979).

Although R-type pyocins were first discovered in *P. aeruginosa*, they have been described in many other bacterial taxa. They have been given many different names such as the xenorhabdicin of *Xenorhabdus nematophilus* (Thaler et al., 1995; Morales-Soto et al., 2012; Thappeta et al., 2020), the carotovoricin of *Pectobacterium carotovorum* (Nguyen et al., 1999), the enterocoliticin of *Yersinia enterocolitica* (Strauch et al., 2001), the fonticin of *Pragia fontium* (Smarda and Benada, 2005; Látrová et al., 2023), the aquaticin from *Budvicia aquatic* (Smarda and Benada, 2005), the kosakonicin of *Kosakonia radicincitans* (Patz et al., 2019), the syringacin of *Pseudomonas syringae* (Haag and Vidaver, 1974), the diffocin of *Clostridium difficile* (Schwemmlein et al., 2018), as well as simply phage-like bacteriocin, phage-like pyocin or R-type bacteriocin (Schwinghamer et al., 1973; Gissmann and Lotz, 1974; Brown et al., 1976; Fischer et al., 2012; Gebhart et al., 2012). To simplify the nomenclature, the term “tailocin” was proposed by Gill and Young (2011) and to follow this nomenclature, we will be referring to these specific eCIS particles as R-tailocins hereafter.

R-tailocins are not the only eCISs that have been found in bacteria. A new group of eCISs targeting eukaryotic cells was discovered in the early 2000s. During this period phage tail-like particles were discovered in *Serratia entomophila* and in *Photorhabdus luminescens* and shown to confer entomopathogenic activity to the two insect pathogens (Hurst et al., 2004, 2007; Yang et al., 2006). The sequences of the genes encoding these eCISs, termed Afp (antifeeding prophage) in *S. entomophila* and PVC (Photorhabdus virulence cassette) in *P. luminescens,* were found to share similarity with phage-related genes and their structural similarity to R-tailocins was noted (Hurst et al., 2004, 2007; Yang et al., 2006). Afp variants conferring insecticidal activity were later also discovered in several strains of the insect pathogen *Serratia proteamaculans* (Hurst et al., 2018, 2021). In 2014, another eCIS consisting of arrays of up to 100 phage tail-like structures, called MACs, was discovered in *Pseudoalteromonas luteoviolacea*, which induces the settlement and metamorphosis of the larvae of the marine tubeworm *Hydroides elegans* (Shikuma et al., 2014). The potential of these eCIS to translocate toxic effector proteins, named eCIS-associated toxins (EATs), into target cells has been demonstrated for PVCs and MACs and has also been postulated for Afps (Heymann et al., 2013; Rocchi et al., 2019; Vlisidou et al., 2019; Geller et al., 2021; Wang et al., 2022). Although the R-tailocins, Afps, PVCs and MACs are currently the best characterized eCISs, genomic analyses have identified sequences for thousands more phage tail-related particles that appear to target bacteria as well as eukaryotic cells (Sarris et al., 2014; Chen et al., 2019; Rojas et al., 2020; Geller et al., 2021). Although most of these structures and / or genetic sequences have been studied in Gram-negative bacteria, genomic analysis have also identified them in archaea and Gram-positive bacteria (Sarris et al., 2014; Chen et al., 2019; Geller et al., 2021; Nagakubo et al., 2021; Babar et al., 2022; Le Faou and Boudier, 2022; Casu et al., 2023; Vladimirov et al., 2023). Furthermore, novel CIS particles have been recently discovered, notably the thylakoid-anchored tCIS in cyanobacteria (Weiss et al., 2022), the AlgoCIS in *Algoriphagus machipongonensis* (Xu et al., 2022), and the *Streptomyces* cytosolic CIS^SC^. They appear to remain cytosolic and mediate the death of the producer cell in response to external stress (Casu et al., 2023; Vladimirov et al., 2023), pointing towards the extensive structural and functional diversity that still needs to be explored.

From 1990 up to the present, the interest in these particles has continued, with the visualization of the dynamics of their production, explosive release and killing activity and studies of their regulation (Matsui et al., 1993; Sun et al., 2014; Penterman et al., 2014b; Fernandez et al., 2020; Vacheron et al., 2021; Bronson et al., 2022), their atomic structures and molecular mechanics (Heymann et al., 2013; Ge et al., 2015, 2020; Desfosses et al., 2019; Jiang et al., 2019; Fraser et al., 2021), their role in bacterial competition (Heo et al., 2007; Waite and Curtis, 2009; Dorosky et al., 2017, 2018; Oluyombo et al., 2019; Vacheron et al., 2021; Heiman et al., 2022) and their potential medical use (Scholl and Martin, 2008; Scholl et al., 2009; Ritchie et al., 2011; Redero et al., 2020; Alqahtani et al., 2021; Six et al., 2021; Bhattacharjee et al., 2022) as well as their application as prophylactic or curative treatment against phytopathogens in agriculture (Fernandez et al., 2017; Príncipe et al., 2018; Baltrus et al., 2022). Recent studies have also re-engineered eCIS structures to target new cell types and to deliver new proteins, opening new avenues for the usage of these particles (Scholl, 2017; Bhattacharjee et al., 2022; Jiang et al., 2022; Kreitz et al., 2023).

## Domestication of eCISs

The eCISs are structurally related to bacteriophage tails. This had already been suggested by Jacob when he discovered these particles (Jacob, 1954) and was a common conclusion throughout the studies focusing on their characterization (Ikeda et al., 1964; Ishii et al., 1965; Kageyama et al., 1979; Shinomiya and Shiga, 1979; Shinomiya, 1984; Shinomiya and Ina, 1989). Since then, it has been confirmed that eCISs originate from phages belonging to the *Caudovirales* order and have a common ancestor with *Myoviridae* phages (Nakayama et al., 2000; Sarris et al., 2014). The non-contractile flexible F-type tailocins are related to *Siphoviridae* phages (Nakayama et al., 2000; Saha et al., 2023). Although it has been shown that eCISs are related to phages, there still is little knowledge on how a prophage evolves into an eCIS (Bobay et al., 2014).

**Figure 3:**
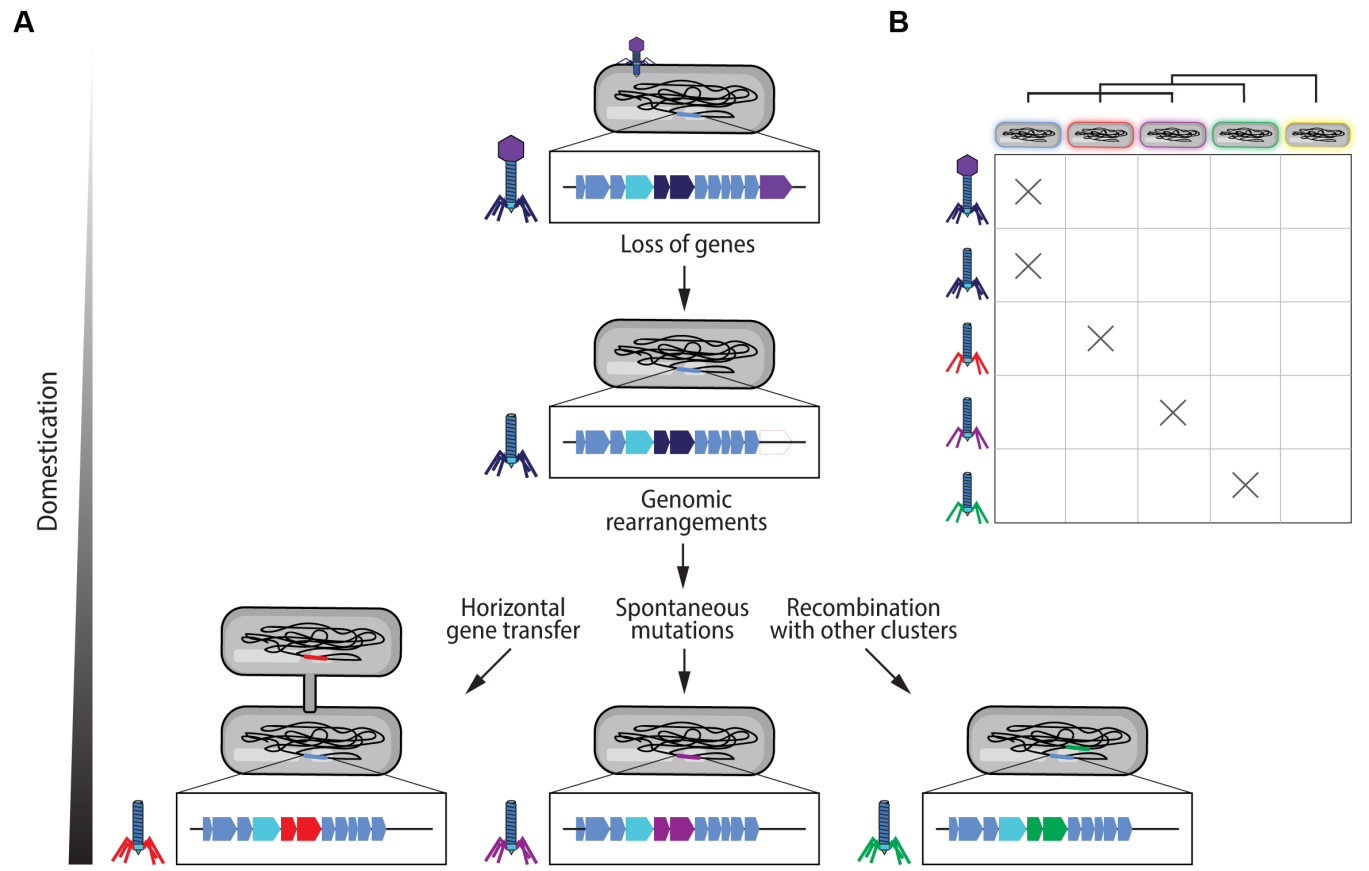
Domestication of prophage gene clusters into extracellular contractile injection systems (eCISs). **(A)** Following the insertion of the genome of a phage into the host bacterial genome, the prophage sequence becomes susceptible to genetic loss giving rise to eCIS loci. Genomic rearrangements and degradation, in addition to spontaneous mutations, can lead to changes in eCIS specificity and loss of certain genes. These clusters can also recombine with other phage-like clusters within the bacterial genome. Horizontal gene transfer can also lead to the acquisition of new structures. These differences are illustrated with the example of tail fibers changes. **(B)** Through these mechanisms, either alone or concomitantly, prophage gene clusters can evolve into new CIS gene clusters with different activity spectra. These changes in spectrum are represented by the different colors of the tail fibers and the lipopolysaccharides coating the different bacteria, where the same color symbolizes a potential interaction between a target bacterium and an eCIS particle. The yellow bacterial cell represents a bacterium that is resistant to all of the viral particles shown, but could be a potential future target that eCIS particles could evolve to attack.

It has been demonstrated that eCISs are not merely degenerated phage particles (Shinomiya and Shiga, 1979). However, several steps from a phage to an efficient eCIS particle can be postulated. Following the insertion of a phage genome into a bacterial genome during its lysogenic cycle, the prophage sequence becomes susceptible to the evolution of the bacterial genome through genomic rearrangements, degradation, and spontaneous mutations (Canchaya et al., 2003; Bobay et al., 2014; Feiner et al., 2015; Salmond and Fineran, 2015; Ofir and Sorek, 2018; Figure 3). For example, the genes affiliated to the head of the phage could be lost, creating a headless phage, in addition, genes determining target specificity, such as those encoding the tail fibers, could become more or differently specialized (Figure 3). Furthermore, since R-tailocin loci in particular are found concurrently with or even nested within prophage sequences, it is thought that there is an important recombination rate between these loci generating new phage tail-like structures (Ghequire and De Mot, 2014; Ghequire et al., 2015; Patz et al., 2019; Vacheron et al., 2021; Figure 3). Additionally, horizontal gene transfer could permit the acquisition of new sequences and genes leading to changes in the structures of eCISs or their activity spectrum as well as the potential acquisition of toxic cargos (Canchaya et al., 2003; Sarris et al., 2014; Figure 3). At present, it remains unclear whether eCISs are generated either by evolution of a prophage sequence, recombination with pre-existing prophages, horizontal gene transfer, or all three (Touchon et al., 2014, 2017; Ghequire et al., 2015; Ghequire and De Mot, 2015; Koonin et al., 2019; Patz et al., 2019; Figure 3). Furthermore, changes in bacterial cell envelope composition, notably in the highly variable O-antigens of LPS cell surface decorations, which are targeted by eCISs, are also thought to drive the evolution of eCIS particles (Carim et al., 2021; Heiman et al., 2022; Figure 3).

Noteworthy, it has been speculated that eCISs could be intermediate structures towards the more complex T6SSs (Büttner et al., 2016; Böck et al., 2017). However, eCISs and T6SSs often co-occur in bacterial cells and are likely to have complementary activities, as the T6SS does not induce the lysis of the producing cell but is only efficient for contact-dependent altercations, while eCISs are released through the lysis of the producing cell but can affect more distant targets.

## Architectures of eCISs

The common ancestry of eCISs with phages is obvious when comparing their genetic organization, especially in the genes encoding the structural components for the eCIS particles (for a more detailed review of eCIS structures, see Taylor et al., 2018). As previously described, eCISs are particles that resemble “headless phages” formed out of a multi-subunit helicoidal sheath encapsulating a tube that is itself composed of multiple subunits (Ishii et al., 1965; Figure 1). The repeating unit of R-tailocin sheaths is a single protein. In many eCISs that target eukaryotic cells such as the Afp and PVC structures, the sheath contains multiple copies of two or three highly similar proteins (Ge et al., 2015; Desfosses et al., 2019; Jiang et al., 2019; Figure 1). Following genomic analysis, some eCIS gene clusters were even found to encode three different sheath subunits (Sarris et al., 2014). The sheath and the tube form the contractile part of the eCIS machine. In some eCISs, this hollow tube can transport toxic effectors named EATs (eCIS-associated toxins), which could target nucleic acids, peptidoglycan or actin (Geller et al., 2021). The EAT effectors Pnf and Pdp1 harbored by PVCs induce destabilization of the actin cytoskeleton and disrupt dNTP pools of the targeted cells, respectively (Yang et al., 2006; Wang et al., 2022), while in the Afps, toxic cargo proteins have been proposed to be the factors leading to feeding cessation in New Zealand grass grub larvae (Hurst et al., 2004; Heymann et al., 2013). An EAT with nuclease activity (Pne1) that kills eukaryotic cell lines on delivery has also been found in MACs (Rocchi et al., 2019), in addition to an effector protein (MifA) responsible for inducing metamorphosis in tubeworm larvae (Ericson et al., 2019).

The contractile tail of eCISs is attached to a baseplate to which are bound a needle-like structure called the spike as well as the tail fibers (Figure 1). Some eCISs have a proline-alanine–alanine-arginine (PAAR) repeat domain on the baseplate spike that is capable of binding the toxic effectors (Shneider et al., 2013; Sarris et al., 2014; Vlisidou et al., 2019). These PAAR domains are absent in R-tailocins, which are not known to harbor effectors (Sarris et al., 2014). Six tail fibers at the periphery of the baseplate mediate target recognition (Ishii et al., 1965; Michel-Briand and Baysse, 2002; Ge et al., 2015; Ghequire et al., 2015; Ghequire and De Mot, 2015; Scholl, 2017; Buth et al., 2018; Dorosky et al., 2018; Schwemmlein et al., 2018; Desfosses et al., 2019). The tail fibers associated with eCISs that target eukaryotic cells have been found to be more similar to *Adenoviridae* fibers, while those associated with eCISs that bind bacteria have higher similarity with phages (Hurst et al., 2004, 2007, 2018; Geller et al., 2021). Cryo-electron microscopy of different eCISs structures shows that the fibers, formed out of three polypeptides, are folded toward the sheath until the eCIS structure reaches a target, at which time the fibers unfold and cause the particle to become perpendicular to the cell surface of the target (Jiang et al., 2019; Ge et al., 2020; Fraser et al., 2021). Once irreversibly bound, the tail fibers initiate the signal for sheath contraction (Jiang et al., 2019; Ge et al., 2020; Fraser et al., 2021).

The first step in the assembly of eCIS structures is the recruitment of the baseplate subunits around the spike (Jiang et al., 2019). Then, the tube subunits polymerize from the baseplate (Jiang et al., 2019). As additional subunits are added to the baseplate to further stabilize it, the tail fibers also bind to the baseplate (Jiang et al., 2019). The tail tape-measure regulates the correct length of the tube by scaffolding the tube (Jiang et al., 2019). Once the optimal length is reached, the cap [also named collar; (Ge et al., 2015; Jiang et al., 2019)] terminates the tube elongation and permits the stabilization of the structure (Rybakova et al., 2013) and the sheath is added to finalize the assembly of the structure (Jiang et al., 2019).

The clusters encoding eCISs can be highly conserved not only in content but also in genomic location. Tailocins (both F- and R-type) belonging to *P. aeruginosa* strains are located in the intergenic region of the tryptophan operon between the *trpE* and *trpGCD* genes, while those belonging to other *Pseudomonas* strains, such as those of the *P. fluorescens* clade, are located between the *mutS* and *cinA* genes (Ghequire and De Mot, 2014; Ghequire et al., 2015; Scholl, 2017; Blasco et al., 2023). The similarities between R-tailocins from *P. aeruginosa* strains have led to a further classification into five different subtypes (R1-R5) according to their structures and activity spectrum (Ghequire and De Mot, 2014).

Although there are many similarities in the organization of the gene clusters and the basic structural components of eCISs, some eCISs differ from the others. In addition, although most eCISs are encoded on the chromosome of the producing bacterium, some, such as the Afps of *S. entomophila*, can be found encoded on plasmids (Glare et al., 1996; Hurst et al., 2004; Chen et al., 2019; Geller et al., 2021). There are also differences in the overall structure of the devices. This is most obvious for MACs, which differ from all other eCISs in that they are composed of arrays of approximately 100 individual contractile phage tail-like structures linked together by their tail fibers (Shikuma et al., 2014; Figure 1). Their structure suggests a “cooperative firing” of the individual phage tail-like particles (Shikuma et al., 2014).

## Production, release, and action mechanism

**Figure 4:**
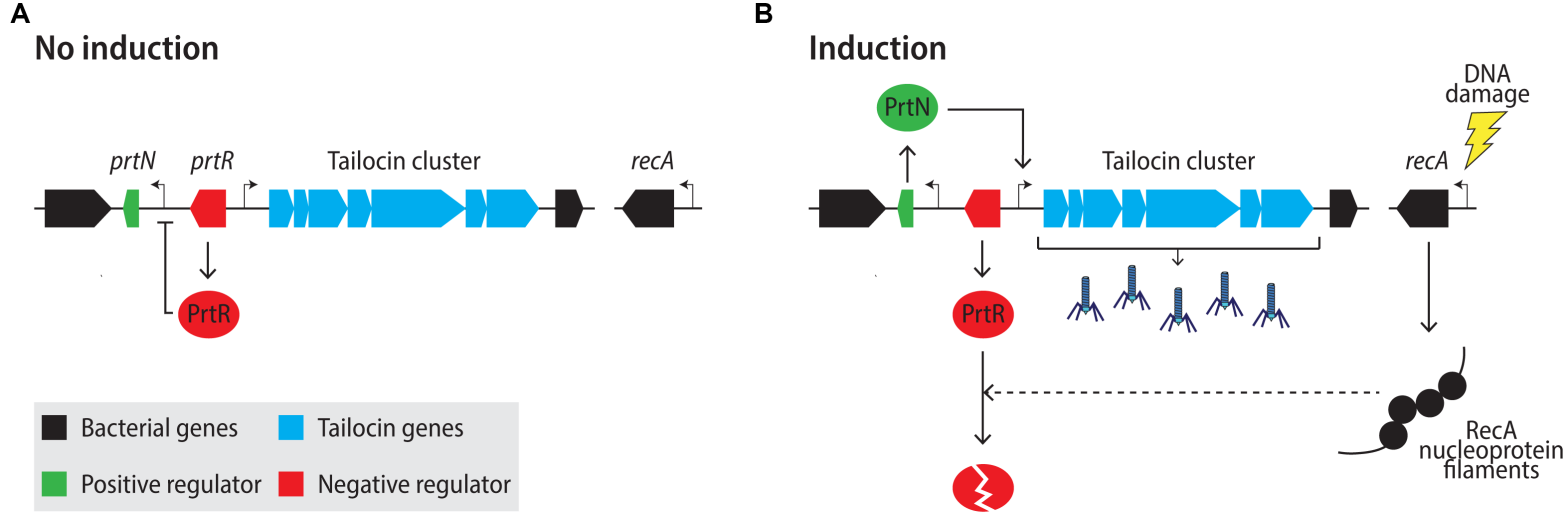
Model of R-tailocin regulation in *Pseudomonas aeruginosa* under non-inducing **(A)** and inducing **(B)** conditions. **(A)** Under non-inducing conditions, the tailocin locus-associated repressor PrtR inhibits the expression of the tailocin activator PrtN. **(B)** Upon DNA damage, RecA forms nucleoprotein filaments on single-stranded DNA, which stimulates the autocleavage of the PrtR repressor, resulting in the derepression of the activator PrtN and the expression of the genes within the tailocin cluster. Sharp arrows represent activation of gene expression, the blunt-arrow represent inhibition of gene expression and the dashed-line arrow represents degradation.

**Figure 5:**
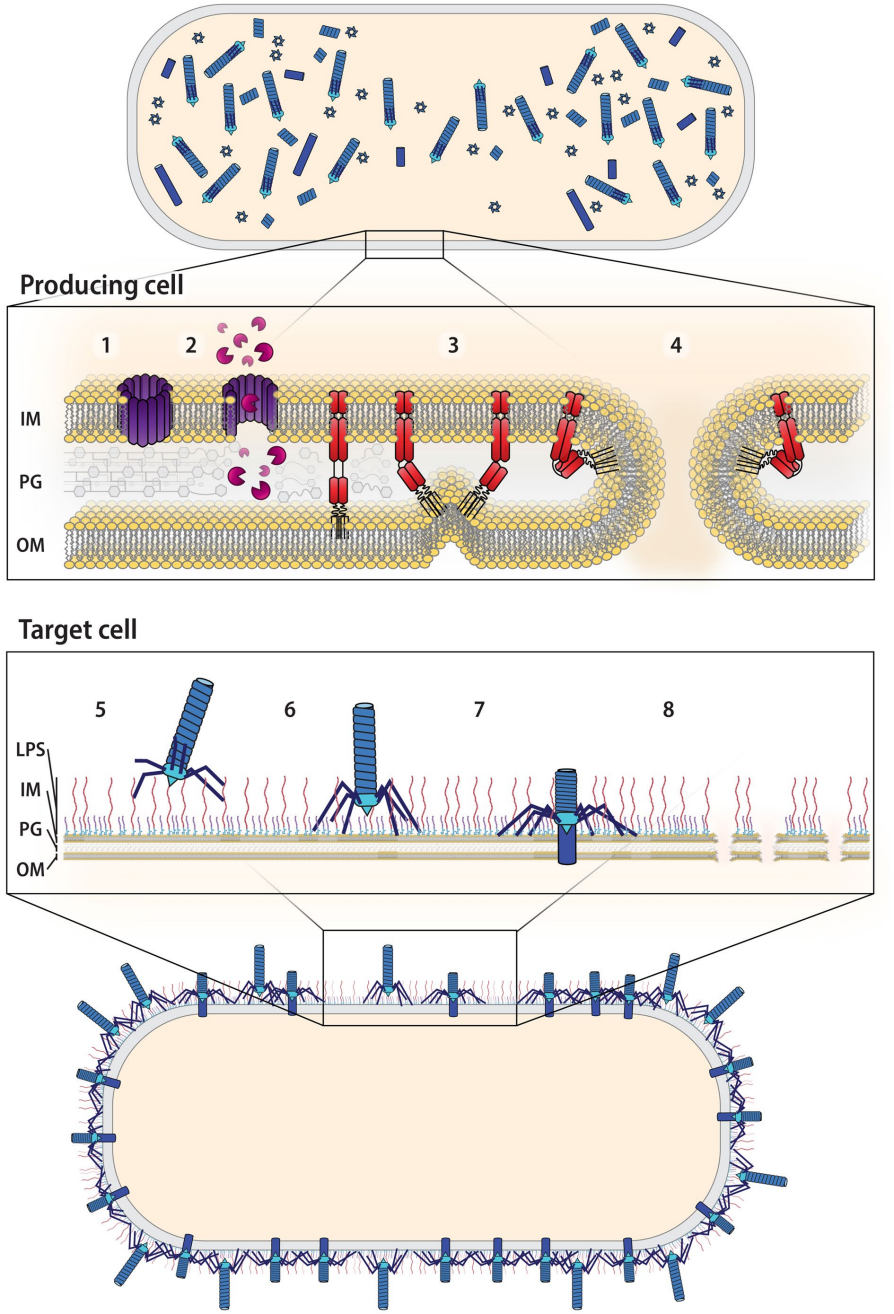
Overview of R-tailocin production up to killing the target cell. Following induction by a stress factor, R-tailocins are assembled in the cytoplasm of the producing cell. 1. Holins, also encoded by the same gene cluster as R-tailocins, will accumulate on the inside of the inner membrane (IM) of the producing cell and form pores. 2. These pores will permit the passage of lysins, also encoded by the R-tailocin gene cluster that will degrade the peptidoglycan (PG) layer. 3. Spanins will fuse the outer membrane (OM) to the inner membrane. 4. This will rupture the membrane, resulting in the explosive lysis of the producing cell and the release of the R-tailocins into the environment. 5. The R-tailocins will precisely recognize a target cell through their tail fibers that will bind to distinct cell surface decorations, specifically the O-antigens of the lipopolysaccharides (LPS). 6. Once the target is recognized, the R-tailocin will attach to the surface of the target cell. 7. The R-tailocin sheath will contract and push the tube through the membrane of the target. 8. The accumulated action of multiple R-tailocins will lead to the lysis and death of the target cell.

As the production and release of eCISs has been extensively studied in R-tailocins, we will review what is known about these particular structures. External stresses that provoke DNA damage and trigger the bacterial SOS response are known to activate the production of tailocins, as they also elicit the lytic cycle of prophages (Howard-Varona et al., 2017; Scholl, 2017). These factors include UVs (Lwoff et al., 1950), H_2_O_2_ (Lwoff, 1952; Ackermann and DuBrow, 1987; Banks et al., 2003; Łoś et al., 2010) as well as certain DNA-damaging antibiotics such as mitomycin C (Otsuji et al., 1959; Ackermann and DuBrow, 1987) or ciprofloxacin (Sun et al., 2014; Penterman et al., 2014b). The most commonly used inducers in laboratory practice are UVs (Lwoff et al., 1950) and mitomycin C (Kageyama and Egami, 1962). The regulatory mechanisms controlling the activation of tailocin production during the SOS-response have been studied in some detail in *P. aeruginosa*. Upon DNA damage, RecA in *P. aeruginosa* will polymerize on single-stranded DNA leading to the formation of nucleoprotein filaments that will stimulate the autocleavage of the repressor LexA, which in turn will induce the expression of the SOS system (Cirz et al., 2006; Penterman et al., 2014b; Figure 4). Similarly, RecA will mediate the autoproteolytic cleavage of the structurally related, tailocin locus-associated repressor PrtR that in conditions lacking stress will block the expression of the tailocin locus-specific activator PrtN (Matsui et al., 1993; Ghequire and De Mot, 2014; Penterman et al., 2014b). Derepressed PrtN will stimulate the expression of the tailocin genes within the cluster (Matsui et al., 1993; Ghequire and De Mot, 2014; Penterman et al., 2014b). Once assembled, R-tailocins will accumulate in the cytoplasm prior to lysis (Figure 5). R-tailocins have been shown in environmental *Pseudomonas* to be assembled at the center of the cell and migrate to the poles of the cell prior to lysis (Dar et al., 2021; Vacheron et al., 2021).

In addition to regulating the expression of the structural genes, PrtR and PrtN also coordinate the expression of the genes that mediate the lysis of the producing cell and thus the release of the R-tailocins into the cellular environment (Matsui et al., 1993). In general, three main types of enzymes coordinate the lysis of the bacterial cell: holins, lysins, and spanins (Rajaure et al., 2015; Cahill and Young, 2019). First, holins accumulate at the inner membrane where they will form pores (Figure 5; Young, 2014; Saier et al., 2015). This will permeabilize the inner membrane and permit the passage of lysins, which degrade the peptidoglycan layer (Figure 5). Finally, spanins contribute to cell lysis through the rupture of the outer membrane by fusing it to the inner membrane (Ghequire and De Mot, 2014; Ghequire et al., 2015; Rajaure et al., 2015; Yao et al., 2017; Kongari et al., 2018; Vacheron et al., 2021; Figure 5). The combined action of these enzymes causes the membrane of the producing cell to rupture and form a typical spheroblast structure before explosively lysing and projecting R-tailocins into the environment (Ghequire and De Mot, 2015; Scholl, 2017; Dorosky et al., 2018; Vacheron et al., 2021). Therefore, the production of R-tailocins is quite costly as a cell must lyse and die to release these particles (Dorosky et al., 2017; Carim et al., 2021; Vacheron et al., 2021; Heiman et al., 2022).

Once released, R-tailocins are thought to be projected tens of micrometers away from the originating cell into the environment where they can reach target cells (Vacheron et al., 2021). R-tailocins will interact with their targets through their tail fibers, which will bind to cell surface receptors, specifically the variable O-antigen components of the LPS (Figure 5; Ikeda and Egami, 1969, 1973; Meadow and Wells, 1978; Köhler et al., 2010; Carim et al., 2021; Heiman et al., 2022). Although LPS has been described as the only known receptor for R-tailocins (Ikeda and Egami, 1969, 1973; Meadow and Wells, 1978; Köhler et al., 2010; Carim et al., 2021; Heiman et al., 2022), it is possible that these particles are able to bind to other cell surface components, as phages are known to bind to other structures besides LPS, such as membrane-anchored proteins (Nobrega et al., 2018). Sheath contraction then is triggered by the binding of multiple tail fibers to their receptors (Ge et al., 2020; Fraser et al., 2021) and the energy needed for the contraction is thought to be stored in the extended state during assembly (Ge et al., 2020; Fraser et al., 2021). This action will push the iron-atom reinforced tube tip through the membrane of the target cell (Ge et al., 2020; Fraser et al., 2021; Figure 5). The consequence of this action remains uncertain, with reports of changes in membrane permeability (Uratani and Kageyama, 1977; Uratani, 1982), loss of membrane potential, increased potassium efflux, or inhibition of proline uptake (Uratani and Hoshino, 1984; Fernandez et al., 2017; Scholl, 2017; Patz et al., 2019; Látrová et al., 2023). However, R-tailocins do act on the target, and the final outcome is membrane disruption and the death of the target cell (Figure 5). Although early studies determined that in the case of R-tailocin (R-pyocin) from *P. aeruginosa*, in principle, one particle is sufficient to kill a bacterial cell (Kageyama et al., 1964), it remains to be seen whether similar killing activities apply to other R-tailocins or eCISs in general.

The production of certain eCISs may be regulated differently from the well-investigated PrtN-PrtR regulation described above (Figure 4). Indeed, in non-pathogenic plant-associated *Pseudomonas* strains, only an ortholog of the *prtR* gene from *P. aeruginosa* was found, but none for *prtN* (Ghequire et al., 2015; Fernandez et al., 2020; Vacheron et al., 2021). Some eCIS loci also completely lack the lysis cassette as well as conserved regulatory genes (Sarris et al., 2014). Thus, it remains unclear at present how these clusters are regulated and which elicitors induce their production, although alternative regulatory pathways that induce the production of R-tailocins appear to exist (Baggett et al., 2021).

## Ecological and evolutionary role of eCISs

Extracellular CISs are widespread throughout all bacteria (Sarris et al., 2014; Ghequire and De Mot, 2015; Chen et al., 2019; Patz et al., 2019; Rojas et al., 2020; Geller et al., 2021), implying important ecological and evolutionary roles for these particles, which may differ depending on their specific activities. In the case of R-tailocin type eCISs these devices target, perforate, and kill bacterial cells and therefore play an important role in interbacterial interactions. Contrary to R-tailocins, AFP-, PVC- and MAC-type eCIS can carry effector proteins with toxic activities that they inject into eukaryotic cells and probably also into bacterial cells (Geller et al., 2021), enabling them with specialized functionality in host interactions.

### Effect on niche colonization

When R-tailocin type eCISs were first discovered, they were induced, extracted and then tested on a small array of strains (Jacob, 1954; Kageyama and Egami, 1962). Most often, these and subsequent studies focused on a pinpointed group of bacterial strains of interest. However, recent studies have expanded the phylogenetic and ecological relationships of the bacteria tested. R-tailocin type eCIS have proven to be highly specialized weapons, generally targeting different strains of the same species or strains of closely related species. As an example in environmental *Pseudomonas*, the R-tailocins extracted from the plant root-colonizing strain *Pseudomonas protegens* CHA0 specifically targets strains belonging to the same species that share more than 97% of genomic identity (Vacheron et al., 2021). Other R-tailocins such as those from strains of plant-associated *Pseudomonas chlororaphis*, *Pseudomonas fluorescens*, *Pseudomonas syringae*, or *Pseudomonas putida* exhibited broader killing spectra, affecting phylogenetically more distant strains belonging to other subgroups or related species (Fischer et al., 2012; Ghequire et al., 2015; Hockett et al., 2015; Dorosky et al., 2017, 2018). For example, the tailocin of *Pseudomonas fluorescens* SF4c can target other strains belonging to the *P. fluorescens* subgroup as well as strains belonging to the *Pseudomonas putida*, the *Pseudomonas corrugata* or the *Pseudomonas syringae* subgroups (Fischer et al., 2012). Similar variability in the intra- and interspecific killing spectra of R-tailocins has been described for clinical isolates of human pathogens such as *P. aeruginosa*, *Clostridium difficile*, or *Burkholderia cenocepacia* (Köhler et al., 2010; Gebhart et al., 2012; Yao et al., 2017; Oluyombo et al., 2019; Blasco et al., 2023). Some R-tailocin type eCIS can even impact strains belonging to different bacterial genera, as has been demonstrated for several *Pseudomonas* R-tailocins targeting leaf pathogenic *Xanthomonas* strains (Fernandez et al., 2017; Dorosky et al., 2018; Príncipe et al., 2018; Weaver et al., 2022). The target specificity and consequently the activity spectra of R-tailocins are ruled by highly specific interactions of the tailocin tail fibers with bacterial LPS cell surface decorations. Depending on the particular O-antigen decoration, the LPS can act either as a receptor for or as a shield against specific tailocins, thus determining the outcome of interbacterial competitions in host- or abiotic environment-associated biofilms (Köhler et al., 2010; Penterman et al., 2014a; Carim et al., 2021; Heiman et al., 2022).

**Figure 6:**
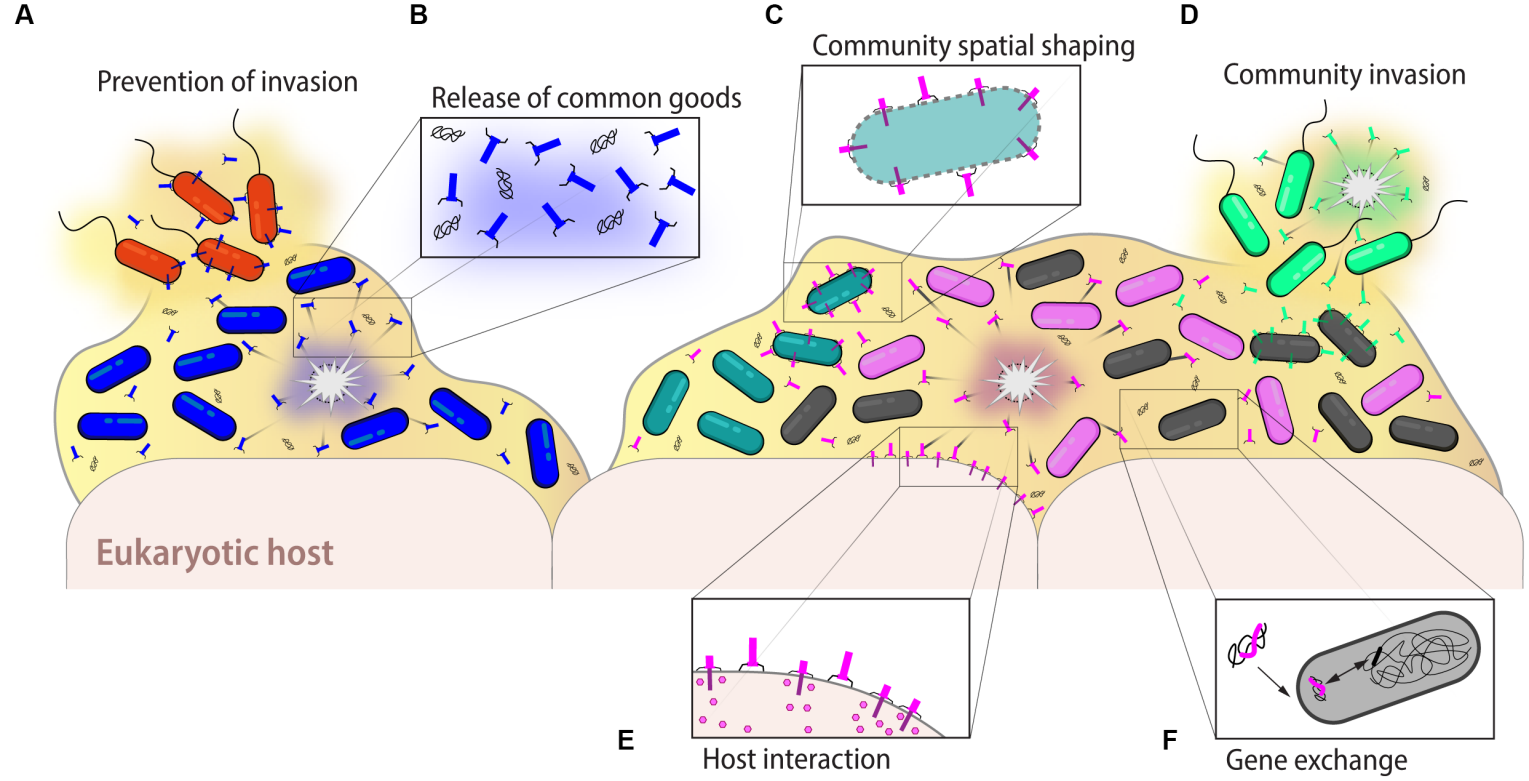
Ecological and evolutionary roles of eCISs. Extracellular CISs can have many different roles impacting bacterial communities. **(A)** In monoclonal as well as polyclonal bacterial populations, eCISs may ward off potential invaders, notably against phylogenetically related bacteria to exclude competitors that target the same niche and nutritional environment. **(B)** Lysis of the eCIS-producing cells releases not only the particles themselves, but also nutrients and DNA, which become a common good accessible to the entire community. The stochastic release of eCISs also permits to maintain the population in a preconditioned state of alert, preventing the invasion by other strains. **(C)** Furthermore, in multi-species bacterial populations, eCISs help to shape the community for example by maintaining the spatialization of different strains within the environment. **(D)** Conversely, eCISs may also help in the colonization of a new niche by displacing established strains. **(E)** In host-associated strains, eCISs could help mediate the interaction between the bacterial population and the host, depicted here with the interaction between eCISs and eukaryotic cells shown below the bacterial biofilm. **(F)** Finally, the eDNA available following the lysis of a producing strain may benefit bacterial evolution through horizontal gene transfer permitting for instance the exchange of different eCIS genes, creating new particles with different activities or activity spectra. Examples and further details are given in the text.

Because of the specialized effect of R-tailocins on other bacteria, most studies have examined their role as weapons used to specifically combat competitors, allowing bacteria to colonize a niche of interest (Figure 6D). Once a strain has achieved niche dominance, tailocins would help to maintain that status. Indeed, R-tailocins produced by pathogenic *P. aeruginosa* strains shape ecological interactions in cystic fibrosis patients by displacing other *P. aeruginosa* strains and influencing strain dominance (Köhler et al., 2010; Oluyombo et al., 2019; Mei et al., 2021). In the wheat rhizosphere (i.e., the soil surrounding wheat roots), a complex environment that is prone to competition, R-tailocins were found to be important for bacterial persistence, as R-tailocin mutants were not as abundant as the wild-type strain (Dorosky et al., 2017, 2018). Tailocin clusters may encode multiple R-tailocins or harbor multiple tail fiber genes, which could specify different spectra of activity leading to a broader target range (Ghequire et al., 2015; Dorosky et al., 2017, 2018; Patz et al., 2019; Vacheron et al., 2021). Moreover, R-tailocins may evolve rapidly as suggested in *Pseudomonas syringae* strains where the recombination of two genes encoding a receptor binding protein (tail fiber protein) and a chaperone was sufficient to alter the activity spectrum of a tailocin (Baltrus et al., 2019), leading to the hypothesis that they could quickly adapt to other targets. Modification of tail fibers upon DNA inversion and module exchange between strains also resulted in altered host range specificity of R-tailocins and competitiveness in strains of *Xenorhabdus bovienii* and *Pectobacterium carotovorum* (Nguyen et al., 2001; Ciezki et al., 2017). Thus, bacteria appear to evolve the activity spectra of their R-tailocin type eCISs in response to environmental pressures to adopt their use in different interactions.

### Effect on host interactions

Although initially eCISs were only shown to affect prokaryotic cells, some, similar to T6SSs, can also interact with eukaryotic targets, resulting in beneficial or deleterious effects on their hosts (Figure 6E). As an example of where eCISs are of benefit for the target eukaryote host, MACs released by *P. luteoviolacea* induce the settlement and metamorphosis of larvae of the marine tubeworm *Hydroides elegans* into the adult stage after interaction with these arrays, which consist of approximately 100 eCISs that expand to a star-like structure upon release (Huang et al., 2012; Shikuma et al., 2014, 2016; Cavalcanti et al., 2020; Hadfield et al., 2021). MACs initiate the loss of the larval cilia and induce genes involved in tissue metamorphosis, innate immunity, and MAPK signaling (Shikuma et al., 2016). A MAC-transported protein effector termed metamorphosis-inducing factor 1 (Mif1) is sufficient to stimulate larval metamorphosis (Ericson et al., 2019). Although this appears to be a beneficial mechanism for the host *H. elegans*, it has been suggested that for the producer bacterium *P. luteoviolacea* this may be an anti-protist mechanism to counteract ciliated predators (Hadfield et al., 2021). Interestingly, MACs cargo a second effector with nuclease activity, which is toxic to several eukaryotic cell lines but has no effect on *Hydroides* larvae and has been suggested to help the *P. luteoviolacea* bacteria cope with protozoan predators (Rocchi et al., 2019; Cavalcanti et al., 2020). Extracellular CISs can also contribute to the virulence of pathogenic bacteria. PVCs and Afps are important pathogenicity determinants of insect-pathogenic *Photorhabdus* and *Serratia* species, respectively, by delivering different toxic effectors into their target eukaryotic cells (Hurst et al., 2004, 2007, 2018; Yang et al., 2006; Rybakova et al., 2013; Vlisidou et al., 2019; Wang et al., 2022). Toxic effectors translocated by eCIS may not only affect invertebrate hosts such as worms and insects, but there are also first examples of eCIS effectors affecting vertebrate hosts. An eCIS-associated toxic effector of the fish pathogen *Yersinia ruckeri*, related to the Afp 18 toxin that is predicted to be loaded by *Serratia* Afps, was found to severely impair the early development of zebrafish embryos by exhibiting glycosyltransferase activity that disrupts RhoA GTPase-driven actin filament organization, ultimately leading to embryo death (Jank et al., 2015).

Furthermore, eCISs particles can have multi-layered effects in bacterial host interactions. For example, *Xenorhabdus nematophila* symbionts of the entomopathogenic nematode *Steinernema carpocapsae* use their R-tailocin type eCISs to outcompete *Photorhabdus luminescens* permitting the reproduction of the nematodes and the exploitation of the insect host (Morales-Soto and Forst, 2011). However *X. nematophila* eCIS mutants were no longer able to affect *P. luminescens*, which inhibited the reproduction of the nematode *S. carpocapsae* (Thappeta et al., 2020). Conversely, in the human gut, a potential beneficial role of eCISs has been suggested based on a higher number of *Bacterodiales* sequences specifying a novel type of eCIS related to Afps, PVCs and MACs in the metagenomes of healthy individuals compared to individuals with inflammatory bowel syndromes such as Crohn’s disease or ulcerative colitis (Rojas et al., 2020). Thus, eCISs are important not only for interactions with other bacteria, but also with the eukaryotic host or with its microbiota, acting as mediators to create beneficial or pathogenic relationships that may be essential for the symbiosis with the host.

### Shaping microbial communities

An invading strain could use eCISs against an established multi-species biofilm to disrupt it and gain access to the ecological niche (Figures 6C,D). Some epidemic strains of *P. aeruginosa* infecting cystic fibrosis patients have proven to be very efficient in their patient colonization and dominance, as they were able to displace the resident strains and prevent colonization by new strains (McCallum et al., 2001; Carter et al., 2010; Oluyombo et al., 2019). It has been suggested that tailocins and phages may play a role in their competitiveness (Burns et al., 2015; Lemieux et al., 2016; Oluyombo et al., 2019). Indeed, R-tailocin mutants could cohabit while their parental strains competed (Oluyombo et al., 2019). Furthermore, when concentrates of purified R-tailocins from one *P. aeruginosa* strain were applied to the established biofilm of a sensitive competitor strain, a significant antimicrobial effect was observed, suggesting that strain dominance could be linked to R-tailocin production (Oluyombo et al., 2019). Conversely, at low concentrations, R-tailocins induced biofilm formation in *P. aeruginosa* (Oliveira et al., 2015). Indeed, the growth of a strain that is sensitive to the R-tailocins of another strain was inhibited, but biofilm formation was induced upon contact with the R-tailocins triggering a population response to the threat of cellular damage (Oliveira et al., 2015). This shows that R-tailocins, and eCIS in general, may have different effects on microbial communities depending on their concentration. It would therefore be interesting to study the effect of increasing concentrations of purified eCISs on already established microbial communities.

Phages have been shown to play a crucial role in shaping microbial communities. Many models have been proposed to describe their predator–prey dynamics with bacterial hosts and the bacterial population. One of the best known is the “Kill-the-Winner” (KtW) model where phages will kill their most abundant host bacteria, leading to a reduction in their abundance (Thingstad and Lignell, 1997; Thingstad, 2000), thus permitting bacteria with lower abundance to increase their population size. Since, many other models derived from the KtW model have emerged to illustrate the different dynamics between phages and their target bacteria (Knowles et al., 2016; Maslov and Sneppen, 2017). Despite being evolved from phages, it is difficult to infer the role and eco-evolutionary dynamics of CISs from those of phages. Extracellular CISs, unlike phages, do not harbor their own DNA and consequently cannot replicate and multiply on their own. Therefore, they cannot be considered as independent entities. Furthermore, the release of eCISs has been proposed to be a self-sacrificial mechanism of the producing bacterium to protect and benefit the entire population (Vacheron et al., 2021). Thus, their production and release need to be precisely regulated so that enough eCISs are released into the environment to fend off competitors without sacrificing the entire population through suicidal eCIS production. It has been shown that a very small percentage of a bacterial population will naturally (i.e., without induction) produce and release R-tailocins (Dar et al., 2021; Vacheron et al., 2021). Thus, eCISs could be used by bacteria as a mechanism to maintain the cell population in a preconditioned state of alert to compete against potential invaders (Figure 6A). Competitor sensing could then detect an incoming attack and trigger a population-level response that could result in mass cell suicide to release sufficient toxins against lethal invaders (Mavridou et al., 2018; Granato and Foster, 2020). Alternatively, the production and release of eCIS could be a mechanism to gain more space and nutrients (Figures 6B,C) when a population becomes too dense.

Nevertheless, as phages, eCISs may have an important role in the assembly and shaping of bacterial communities in all types of environments. Yet, the dynamics between eCIS production and members of the microbiota have never been studied in depth, *in vivo*, or in an environmental context. Therefore, it would be interesting to investigate these dynamics by looking at the production of eCISs by community members within an already established community to see their effect on the microscale stabilization of the bacterial population and their role in preventing invasion by competitors. Furthermore, the invasion of a new strain could be mimicked by studying the effect of adding eCISs to an already established community and tracking the repercussions on the community composition and diversity. Although eCISs are highly specialized weapons and generally target only specific bacterial strains, the death of the target strain could have drastic ripple effects on the functioning and diversity of a given ecosystem, impacting other microorganisms and potentially the entire host that would normally interact with that target strain.

### Influencing bacterial evolution

Extracellular CISs, like phages, could also have a role in increasing bacterial genetic diversity and evolution (Figure 6F). The Red Queen hypothesis states that predator (here, an eCIS-producing bacterium) and prey (here, a target bacterium) will continuously counter-evolve to attack or escape, respectively, the other (Van Valen, 1973). In this predator–prey dynamic, tail fibers are a key factor as they are responsible for target recognition and have high diversity compared to the other structural parts of the eCISs, as documented in particular for the R-tailocins (Ghequire and De Mot, 2014; Ghequire et al., 2015; Baltrus et al., 2019; Patz et al., 2019; Vacheron et al., 2021). Furthermore, it has been suggested that multiple tail fiber genes harbored in some tailocin gene clusters may increase the spectrum of activity (Dorosky et al., 2018; Patz et al., 2019). In addition, it has also been shown for phages that they can recombine with unrelated phages to increase their activity spectra (Moura de Sousa et al., 2021), and eCISs in general may do the same with other CIS structures as well as with phages. On the bacterial side, it is known that the genes encoding cell surface components such as LPS O-antigens are subjected to higher mutation rates than other genes (Salaün and Saunders, 2006; Maldonado et al., 2016; Matic, 2016). The changes in these structures, tail fibers and receptors, respectively, on both the attacking eCISs and the bacterial target can lead to differences in bacterial fitness by altering the activity spectra of the eCISs as well as the respective susceptibility of cell surface components of the bacterium, which in turn will affect the way the bacterium will interact with its environment (Kupferschmied et al., 2016; Carim et al., 2021; Heiman et al., 2022).

Furthermore, as it was recently discovered that some EATs encoded in eCISs clusters could be toxic to bacteria (Geller et al., 2021). New mechanisms may emerge to counteract the EATs injected into target cells, which could lead to the discovery of new defense systems or toxin-antitoxin mechanisms. In T6SSs, antibacterial effectors are often encoded adjacent to genes encoding immunity proteins that inactivate the effectors to protect the producer cell (Hersch et al., 2020). However, no similar immunity proteins against antibacterial EATs have been identified so far and it is currently unclear if such immunity systems exist (Geller et al., 2021). Bacteria have evolved other mechanisms granting them protection against T6SS effectors such as envelope stress responses, the production of reactive oxygen species, physical barriers such as exopolysaccharide layers, or general bacterial stress responses (Hersch et al., 2020) that could potentially also be mechanisms used to protect them from any eCIS. Finally, sensing the attack of a competitor could be a way to trigger eCIS production as a means for counter-attack. In some *P. aeruginosa*, the attack of a competitor’s T6SS triggers a retaliation by its own dormant T6SS, leading to a “duel” between the strains (Basler et al., 2013; Hersch et al., 2020).

In addition to causing changes through arms race dynamics, eCISs also cause the lysis of the producing cells as well as the target, thereby contributing to the release of nutrients and DNA into the environment (Turnbull et al., 2016; Vacheron et al., 2021; Toyofuku et al., 2023; Figure 6B). The availability of extracellular DNA (eDNA) contributes to the possibility of horizontal gene transfer within a community (Weinbauer and Rassoulzadegan, 2004; Nanda et al., 2015; Toyofuku et al., 2023). In the naturally competent *Vibrio cholerae*, the T6SS is co-regulated with the DNA uptake machinery (Borgeaud et al., 2015). This mechanism allows the attacking cells to release the DNA from their targets and gain new functions accessible through horizontal gene transfer (Borgeaud et al., 2015; Metzger et al., 2016; Veening and Blokesch, 2017; Matthey et al., 2019). As eCISs are thought to be the intermediate between phages and the T6SS (Büttner et al., 2016; Böck et al., 2017), it could be speculated that this mechanism of purposeful eDNA uptake is also performed through eCIS-induced lysis. However, further studies are required to confirm this relationship.

## Biotechnological applications of eCISs

The usage of eCISs for potential biotechnological applications is not a recent concept. These particles were already used in the 1970s as a tool for *P. aeruginosa* typing or “fingerprinting” to better identify, investigate and control outbreaks (Farmer and Herman, 1969; Rose et al., 1971; Edmonds et al., 1972; Lovrekovich et al., 1972; Jones et al., 1974; Duncan and Booth, 1975; Bruun et al., 1976; Fyfe et al., 1984; Schable et al., 1986). Furthermore, as multi-drug resistant bacterial infections are becoming more frequent and more difficult to treat, the high specificity of eCISs, specifically R-tailocins, has interesting potential for medical applications in the treatment of nosocomial *P. aeruginosa* infections. R-tailocins have been used in murine models infected with *P. aeruginosa* and were found not only to protect the mice from infections, but also to reduce mortality as well as treat the infection (Merrikin and Terry, 1972; Haas et al., 1974; Williams, 1976; Scholl and Martin, 2008; Redero et al., 2020).

Recent studies have placed their efforts in re-engineering eCISs to modify and/or broaden their spectrum of activity, which brings hope for new treatment of multi-drug resistant pathogenic bacteria (Williams et al., 2008; Scholl, 2017; Alqahtani et al., 2022; Bhattacharjee et al., 2022). Indeed, recombinant R-tailocins with modified tail fibers and new target specificities have been used to treat *P. aeruginosa* infected wounds in mice (Alqahtani et al., 2021), to reduce diarrhea and intestinal inflammation caused by enterohemorrhagic *Escherichia coli* in a rabbit model of infection (Ritchie et al., 2011), and to prevent intestinal colonization of mice by the pathogen *Clostridium difficile* (Gebhart et al., 2015). Furthermore, PVC-type eCIS particles known for their ability to transport toxins that target insect cells, were recently repurposed as designable protein delivery system by re-engineering tail fiber domains and by exploiting a signal sequence required for loading of cargo proteins onto the device (Jiang et al., 2022; Kreitz et al., 2023). The engineered eCIS-based protein delivery platform enabled functional delivery of proteins of choice to desired cell types, as demonstrated by delivery of various cargos including Cas9, base editors, and toxins, to human cells (Kreitz et al., 2023) and of an antitumor drug in an effort to treat cancerous mouse models (Jiang et al., 2022).

Biotechnological applications of these particles have also been tested in the agricultural field to assess their usage in the treatment against plant pathogens, creating a potential alternative to commonly used chemical pesticides. An R-tailocin from *P. fluorescens* SF4c targets, besides different *Pseudomonas* strains, also an isolate of the phytopathogen *Xanthomonas vesicatoria* that causes bacterial leaf spot disease in tomato cultivars (Fernandez et al., 2017; Príncipe et al., 2018). Application of the R-tailocin reduced *Xanthomonas* infection on both leaves and tomato fruits (Príncipe et al., 2018). Similarly, prophylactic application of R-tailocins prevented leaf infections caused by *P. syringae* pv. *syringae* B728a in *Nicotiana benthamiana* tobacco plants (Baltrus et al., 2022). The highly selective removal or control of target bacteria within complex microbial communities through the action of phage tail-related particles opens the possibility of using them as a specific tool to achieve microbiome engineering and treat dysbiosis that can lead to disease.

Thus, phage tail-related particles are not only beneficial for the producing bacterial strain, but can also be used for different biotechnological applications. Major challenges will be to identify the specific spectra of different eCISs, their formulation, their application and combination with other means to control pathogens. As more studies explore the structures, mechanisms of action and activity spectra of these particles, the knowledge of how they can be harnessed and altered will also increase to benefit different fields.

## Conclusion

Extracellular CISs are widely distributed phage tail-like structures harbored in a variety of Gram-negative and Gram-positive bacteria and archaea inhabiting diverse environments. Although they are evolutionarily related to phages, their study has revealed a distinct and independent role from that of phages. Extracellular CISs influence microscale interactions between microorganisms. In the context of a microbiota, eCISs may have a complementary role to antimicrobial secondary metabolites, T6SSs and other bacterial weapons helping the establishment of localized micro-communities. Extracellular CISs could influence the interactions within this micro-community and beyond by shaping the structure of the entire microbiota and by influencing spatiotemporal community dynamics. Indeed, eCISs can influence bacterial evolution through arms-race dynamics, leading to simple and complex resistance mechanisms, and by making eDNA and nutrients available as community goods. Extracellular CISs also can also promote interactions with eukaryotic hosts, either beneficially for symbionts or detrimentally for pathogens. Finally, eCISs have great potential for biotechnological applications in different fields ranging from medicine to agriculture.

Nevertheless, relatively little has been done to better understand the ecological role of eCISs in modulating bacterial evolution and ecology. Therefore, future research should investigate the ecological role of eCISs, focusing on the different advantages that these structures may provide to the producer bacteria and their environment, while addressing the challenge of combining meta-omics with spatial biology in complex systems. This will benefit a better understanding of the role of eCISs within bacterial communities and how these devices can be implemented as biotechnological tools.

## Author contributions

CMH: Conceptualization, Writing – original draft, Writing – review & editing, Investigation, Visualization. JV: Conceptualization, Supervision, Writing – original draft, Writing – review & editing, Investigation, Visualization. CK: Conceptualization, Funding acquisition, Project administration, Supervision, Writing – original draft, Writing – review & editing, Investigation, Visualization.
