# Supplementary material for: Evolutionary and ecological role of extracellular contractile injection systems: from threat to weapon
Source: Front Microbiol. 2023 Oct 11;14:1264877. doi: 10.3389/fmicb.2023.1264877 (PMC10598620; doi:10.3389/fmicb.2023.1264877)
Supplement: Supplementary file 1 [file Table_1.docx]

**Supplementary Table 1. Detail of the main findings highlighted in Figure 2.**

| Dates | Finding | References |
| --- | --- | --- |
| 1954 | Discovery of pyocins of *Pseudomonas aeruginosa* | (Jacob, 1954) |
| 1964 | Characterization of rigid R-type pyocins | (Ikeda et al., 1964; Kageyama, 1964; Kageyama et al., 1964) |
| 1964 | First visualization of purified R-type pyocins with electron microscopy | (Kageyama, 1964) |
| 1964 | LPS as pyocin receptors | (Homma and Suzuki, 1964) |
| 1965 | First detailed structural description of R-type pyocins | (Ishii et al., 1965) |
| 1969 | Adsorption of pyocins to target and contraction captured by electron microscopy | (Higerd et al., 1969; Takeya et al., 1969) |
| 1972 | First study using pyocins as a treatment | (Merrikin and Terry, 1972) |
| 1979 | First flexuous F-type pyocin described | (Kuroda and Kageyama, 1979, 1981; Kuroda et al., 1979) |
| 1980 | Tail fibers mediate target recognition | (Ohsumi et al., 1980) |
| 1993 | Investigation of the regulation of pyocin production | (Matsui et al., 1993) |
| 2000 | Viral origin of pyocins confirmed | (Nakayama et al., 2000) |
| 2004 | Discovery of the antifeeding prophage (Afp) of *Serratia entomophila* | (Hurst et al., 2004) |
| 2006 | Discovery of the *Photorhabdus* virulence casette (PVC) | (Yang et al., 2006) |
| 2011 | Term tailocin introduced | (Gill and Young, 2011) |
| 2014 | Discovery of metamorphosis associated contractile structures (MACs) of *Pseudoalteromonas luteoviolacea* | (Shikuma et al., 2014) |
| 2014 | First large-scale phylogenomic study of eCIS loci | (Sarris et al., 2014) |
| 2015 | Atomic structure of extended and contracted R-type pyocin | (Ge et al., 2015) |
| 2018 | Term contractile injection systems (CISs) introduced | (Taylor et al., 2018) |
| 2019 | Atomic structures of extended and contracted PVC and Afp | (Desfosses et al., 2019; Jiang et al., 2019) |
| 2019-2022 | Identification of eCIS-associated toxins (EATs) | (Ericson et al., 2019; Rocchi et al., 2019; Vlisidou et al., 2019; Wang et al., 2022) |
| 2019-2021 | Phylogenomics of eCIS loci and EATs | (Chen et al., 2019; Rojas et al., 2020; Geller et al., 2021) |
| 2020-2021 | Molecular mechanics of target membrane puncturing by R-type pyocin | (Ge et al., 2020; Fraser et al., 2021) |
| 2021 | Visualization of the dynamics of production, explosive release and killing activity of R-tailocins | (Vacheron et al., 2021) |
| 2022-2023 | eCIS engineering for customized protein delivery to new target cells | (Jiang et al., 2022; Kreitz et al., 2023) |
